# Horses and ADHD: the ASTride intervention for cognitive and emotional growth

**DOI:** 10.1186/s13034-025-00990-6

**Published:** 2025-11-21

**Authors:** Anne Helmer, Elaina Delore, Orit Bart

**Affiliations:** 1 Department of Occupational Therapy, School of Health Professions, Gray Faculty of Medical and Health Sciences, Tel Aviv University, Ramat Aviv, 69978 Tel Aviv, Israel; 2 Mount Judeah stable, Tzur Hadassah, Israel; 3 Elwyn Israel, Jerusalem, Israel

**Keywords:** Equine assisted occupational therapy, EAS, ADHD, Hope, Self-efficacy, Executive-functions, Anxiety

## Abstract

**Background:**

This study aimed to evaluate the efficacy of the Attention Skill Training (ASTride) protocol, an Equine-Assisted Occupational Therapy (EAOT) intervention, for children diagnosed with Attention Deficit/Hyperactivity Disorder (ADHD) in enhancing and promoting cognitive and emotional aspects including executive functions, self-efficacy, anxiety and hope perception.

**Methods:**

A prospective cohort study with an Interrupted Time-Series design was conducted. Fifty participants (mean age = 9.51 years, SD = 1.52) were assessed at four time points: baseline, pre-test (following a 12-week waiting period), post-test (after 12 weeks of intervention), and after three-month follow-up. The intervention was administered by a licensed equine assisted occupational therapists, and assessments were conducted by a blinded occupational therapist. Measures included The Behavior Rating Inventory of Executive Function (BRIEF) for executive functions, and the Hope, Child Anxiety Related Emotional Disorders (SCARED), and The New General Self-Efficacy Scale (NGSE) questionnaires for emotional factors.

**Results:**

Significant improvements were found in executive functions, hope perception, and self-efficacy. Anxiety levels significantly decreased post-intervention.

**Conclusion:**

The ASTride intervention showed efficacy in improving executive functions, hope, and self-efficacy, while reducing anxiety in children with ADHD, supporting its potential as a comprehensive therapeutic approach.

*Trial registration* The research is registered at Clinical Trials.gov, identifier number NCT05869253, first registration at 22/5/2023.

## Background

*Attention-Deficit/Hyperactivity Disorder (ADHD)*, a neurodevelopmental disorder marked by symptoms of inattention, hyperactivity, and impulsivity, remains one of the most prevalent pediatric diagnoses. Boys are diagnosed at a rate 2–9 times higher than girls[1], with recent estimates suggesting that ADHD affects around 9.4% of children, highlighting its continued significance as a public health concern [2].

ADHD is associated with chronic impairments in executive functions − high-level cognitive processes primarily linked to the frontal lobes of the brain. Executive functions are crucial for self-regulation, goal-directed behavior, response inhibition, working memory, and cognitive flexibility, enabling individuals to adapt to new situations and manage their behavior effectively [3]. Additionally, ADHD often involves significant psychosocial challenges such as emotional dysregulation, impulsivity, anxiety, depression, aggression [1, 4], low self-esteem, and difficulties in interpersonal relationships [5].

Current best practices for treating ADHD include a combination of pharmacological treatments and evidence-based psychological and behavioral therapies. Stimulant medications such as methylphenidate and amphetamines remain the most common and effective pharmacological treatments for reducing core symptoms [6]. Non-pharmacological interventions often involve cognitive training targeting specific executive functions, utilizing principles of neuroplasticity through intensive, repetitive practice and immediate feedback [7]. However, while these approaches effectively address core symptoms like hyperactivity and inhibition, they do not always translate to improved everyday functioning [8]. Behavioral interventions for ADHD are most effective when they are client-centered and occupation-based, focusing on personal functional goals and strategy acquisition [8]. Although these interventions show promise for improving executive functions and functional skills, they often do not address the full range of psychological difficulties associated with ADHD. Psychological interventions, such as behavioral programs, parental psychoeducation, and expressive therapies, can be beneficial but may not always be occupation-based or focus on strategy acquisition [9]. Recent studies emphasize the need for a more integrated approach that addresses the complex and multifaceted nature of ADHD, combining core symptom management, emotional support, and practical daily functioning [2].

### Feasibility of equine-assisted services for children with ADHD

Equine-Assisted Occupational Therapy (EAOT) combines the therapeutic benefits of equine interaction with traditional occupational therapy techniques [10]. EAOT falls within the therapy domain of Equine-Assisted Services (EAS) which includes other licensed professionals who incorporate horses into practices such as physical therapy, psychotherapy, and speech -language pathology. Recent studies have underscored EAS’s effectiveness in reducing anxiety, improving mood and self-esteem [11], and enhancing attention, EFs, and social skills [12] in individuals with various disabilities, demonstrating the therapy's broad applicability. However, more rigorous and methodologically sound studies are needed to better understand these effects and confirm the findings across different populations and settings [11, 13].

Current research on EAS for children with ADHD is limited, with existing studies varying in sample size, intervention length, and the professionals conducting the interventions. To our knowledge, only one study to date has presented a structured intervention protocol addressing both cognitive and emotional aspects for children with ADHD [14]. Given the demonstrated effectiveness of occupational therapy interventions for children with ADHD and the emerging evidence supporting EAS in enhancing cognitive and emotional functions, this study seeks to further validate ASTride protocol for this population, focusing on enhancing and promoting cognitive and emotional aspects such as executive functions, hope, self-efficacy and anxiety.

## Method

### Participants

The study included 50 participants (mean age = 9.51 years, SD = 1.52), recruited from Mount Judah Stables, an EAS provider to over 300 patients (See Table 1). Out of the 62 participants initially approached, eight declined due to scheduling conflicts, and four were excluded for beginning new pharmacological treatments during the intervention. *Inclusion criteria*: (1) children between 6 and 12 years of age; (2) diagnosis of ADHD from a medical professional based on DSM-5 criteria with or without medication (e.g., psychostimulant medication); (3) general doctors' approval and referral for participation in EAS. *Exclusion criteria*: (1) moderate to severe cognitive impairment; (2) neurological disorders (e.g., epilepsy); (3) children with additional developmental disorders (e.g., Autism, Cerebral Palsy); (4) children due to begin new medical treatment or change existing treatment during intervention; (5) children with severe sensory loss (e.g., blindness).

**Table 1:** Sociodemographic participant characteristics

Characteristic	Participants
	n (%)
*Gender*	
Male	33 (66)
Female	17 (34)
*Medication*	
Yes	24 (48)
No	26 (52)
*Residency*	
City	24 (48)
Community	23 (46)
Town	3 (6)
*Education*	
Special Education	7 (14)
Typical Education	43 (86)

*N* = 50.* M* = Mean; *SD* = Standard Deviation

### Measures

#### ASTride protocol

The ASTride intervention [14] employs a blend of theoretical frameworks to address ADHD-related impairments. It integrates the Multi-Context approach [15], the Cognitive Orientation to Daily Occupational Performance (CO-OP) approach [16], and Sensory Integration (SI) theory [17]. The Multi-Context approach focuses on enhancing strategy use across a variety of activities to promote generalization and improve functional performance. This approach emphasizes training strategies, practice in diverse contexts, and metacognitive skills, gradually shifting intervention activities to mirror real-life challenges[15]. The CO-OP approach complements this by providing a client-centered, performance-based method that uses the Goal-Plan-Do-Check strategy to facilitate skill acquisition, incorporating structured techniques, including visual aids and modeling, to teach basic riding skills and ensure their application in daily activities [16]. SI theory, integral to the ASTride protocol, focuses on how sensory processing impacts behavior and adaptation [17]. In EAOT, riding provides diverse sensory input, such as tactile and vestibular stimuli, with the potential to enhance sensory integration and support overall functioning [18]

Along with its theoretical framework, ASTride is a structured and defined protocol that includes 12 weekly therapeutic sessions, each lasting 45 minutes. It is an intervention administered by an occupational therapist who is an EAS professional. Therapeutic goals are defined at the beginning of the intervention in collaboration with the parents, tailored to meet each child's needs. During the intervention, strategies are learned and practiced within the equine environment, utilizing both mounted and unmounted activities. The learned strategies are later transferred to other environments [14].

Each ASTride session followed a consistent structure that gradually increased in complexity across the 12-week intervention. Sessions began with a brief check-in and review of previously learned strategies, followed by unmounted activities such as grooming, leading, or stable management tasks designed to promote planning, sequencing, and emotional regulation within a functional and motivating context. Mounted activities formed the central component of the intervention and incorporated structured riding exercises tailored to each child’s therapeutic goals, such as following multi-step riding patterns to practice working memory and inhibition, adjusting body position and rein use to support sensory regulation, and applying the Goal–Plan–Do–Check strategy while acquiring new riding skills. Each session concluded with a reflective discussion, in which the therapist guided the child to identify strategies used during the activities and to explore how these could be generalized to everyday contexts such as schoolwork or peer interactions. The 12-session progression moved from basic interaction with the horse and single-step tasks to more complex multi-step patterns and increased demands on executive functions and emotional regulation. Parents were engaged at the beginning and end of each session to reinforce continuity and strategy transfer across environments.

*The Behavior Rating Inventory of Executive Function (BRIEF)* [19]. The BRIEF is an 86-item standardized rating scale completed by parents to assess executive functions in children aged 5–18. It includes eight scales: Inhibit, Shift, Emotional Control, Initiate, Working Memory, Plan–Organize, Organization of Materials, and Monitor. The scales generate two indexes: the Behavior Rating Index (BRI) from the first three scales and the Metacognitive Index (MI) from the rest. The Global Executive Composite (GEC) sums all scores. Items are rated on a Likert scale from 1 (never) to 3 (often). Raw scores are converted to t scores, with 65 or higher considered clinically significant (SD = 10). The BRIEF has established reliability and validity and is validated for measuring executive functions in children with ADHD [19, 20].

*The Children’s Hope Scale (Hope)* [21, 22] comprises 6 statements to which participants respond using a 6-point Likert scale, ranging from 1 (none of the time) to 6 (all of the time). It includes three agency items (e.g., "I think I am doing pretty well") and three pathway items (e.g., "I can think of many ways to get things in life"). The total score of all items forms an overall scale that indicates change. In this study, a Cronbach alpha of .77 was found for the questionnaire.

*The New General Self-Efficacy Scale (NGSE)* [23] is an 11-item, 5-point Likert scale, self-report questionnaire designed to assess an individual's general expectations and perceived abilities to achieve goals and overcome challenges across various situations. The NGSE: (a) predicts specific self-efficacy (SSE) across different contexts and tasks, (b) forecasts general and comprehensive performance outcomes, and (c) mitigates the negative effects of adverse experiences on subsequent SSE. The scale has demonstrated high internal consistency and test-retest reliability.

*The Screen for Child Anxiety Related Emotional Disorders (SCARED)* [24] is a 41-question self-report scale for children and parents that screens DSM-5 anxiety disorders. Responses range from 1("usually true") to 3 ("seldomly true"). A total score of ≥25 indicates an anxiety disorder, while >30 is more definitive. The child and parent SCARED each produce five robust factors: somatic/panic anxiety, general anxiety, separation anxiety, social phobia, and school phobia. The SCARED has demonstrated good reliability and validity [24].

### Procedure

After obtaining ethical approval from the university IRB ethical committee (number 0003949-3), relevant participants on the Mount Judah Stables wait list were approached to participate in the study. Once the consent forms were signed by child and parents, participants were asked to complete a baseline assessment which included the BRIEF, the Hope, the NGSE, and the SCARED. After baseline assessment (time 1), participants proceeded to a 12-week wait period. Following the wait period, assessments were repeated, and directly followed by 12 weeks of ASTride intervention [14] (time 2). Assessments were conducted once again directly at the end of the intervention period (time 3), with follow up (time 4) assessments taking place 12 weeks following the end of intervention and including the BRIEF, NGSE and Hope questionnaires. All assessments were administered by a research assistant blinded to the participant’s stage in the intervention.

### Data analysis

Determination of power analysis, dependent on an 80% probability (delta=2.8) of identifying a significant response with a moderate effect size (Cohen’s d = 0.5) recommends a minimum sample size of 25 children in intervention group.

Statistical analysis was performed using SPSS statistical software Version 27.0, with statistical significance set at *p *< .05. Prior analyzing the data, we compared between children with and without medication and in mainstream education and special education. We found no significant differences in all outcome measures throughout the four phases of the study. Therefore, we referred to participants with and without medication and from the same educational environment as one group. To compare the four assessment time points (baseline, pre-test, post-test, and follow-up), a Repeated-Measures ANOVA was employed while controlling for medication (with and without) and educational environment (mainstream and special education). Post-hoc analyses were conducted using Bonferroni adjustments to identify specific time points where significant differences occurred. Where applicable, effect sizes were calculated to assess the magnitude of the intervention’s impact on outcome measures. Partial eta square was calculated, the values of which are typically referred to as small (0.01), medium (0.06), and large (0.14) [25] .

## Results

Fifty children (33 boys, 17 girls) aged 6–12 participated in the study (mean age 9.51, SD = 1.52). Approximately half of the participants were under pharmacological treatment for ADHD symptoms. Most attended mainstream education system. Demographic characteristics of participants are presented in Table 1.

### Executive functions

A repeated-measures ANOVA was performed to evaluate the effect of time on EFs according to the BRIEF sub scales. Improvement in executive functions, as evidenced by the BRIEF, was found to be statistically significant.

Post-hoc analysis with a Bonferroni adjustment indicated that for all subscales no significant differences were found between time 1 and time 2. However, post-intervention, at time 3, all scales (inhibition, shift, emotional control, initiate, working memory, plan/organize, and monitor) were significantly lower compared to time 2, indicating improvements in executive functions (all p’s< 0.001). Furthermore, Partial Eta Square for all BRIEF subscales were high, indicating clinical improvement in executive functions (see Table 2).

**Table 2:** Changes in executive functions using the BRIEF questionnaire across three time points (N = 50)

	Time 1	Time 2	Time 3			
	M (SD)			F	*p*	Partial Eta Square
Inhibit	63.50 (10.96)	62.62 (12.74)	57.88 (10.92)	9.76	.001	.28
Shift	67.66 (11.67)	65.14 (11.77)	61.64 (11.46)	10.17	.001	.29
Emotional Control	66.42 (10.89)	64.26 (11.47)	56.72 (12.55)	15.07	.001	.38
Initiate	63.10 (10.68)	61.62 (9.64)	56.52 (10.38)	13.65	.001	.36
Working Memory	68.86 (8.02)	67.92 (8.52)	62.34 (8.48)	22.86	.001	.48
Plan/Organize	66 (9.94)	65.58 (8.58)	59.36 (8.67)	20	.001	.45
Organization of Materials	55.92 (9.64)	56.98 (10.50)	51.54 (8.79)	22.88	.001	.48
Monitor	59.92 (9.89)	58.60 (10.53)	53.04 (9.34)	12.66	.001	.34
BRI	67.70 (11.20)	66.02 (11.88)	59.02 (13.05)	12.86	.001	.34
MI	65.56 (8.53)	64.94 (8.42)	58.34 (8.30)	35.63	.001	.59
GEC	67.64 (8.34)	66.24 (8.56)	59.68 (8.46)	35.23	.001	.59

*M = Mean; *SD* = Standard Deviation; BRI = Behavioral Regulation Index; MI = Metacognition Index; GEC = Global Executive Composite

### Emotional functions

A repeated-measures ANOVA was performed to evaluate the effect of time on anxiety according to the SCARED sub scales. Improvement in anxiety levels, as evidenced by the SCARED, was found to be statistically significant (all *p*’s<0.001). Post-hoc analysis with a Bonferroni adjustment indicated that, for all subscales, no significant differences were found between time 1 and time 2. However, post-intervention, at time 3, all scales (all *p*’s< 0.001) except school avoidance (*p* < .627) were significantly lower compared to time 2. Partial Eta Square was high as well, indicating a clinical improvement in anxiety level post-intervention. See Tables 3, 4.

**Table 3:** Changes in Hope and Self-efficacy across three time points (N = 50)

	Time 1	Time 2	Time 3			
	M (SD)			F	*p*	Partial Eta Square
Hope^1^	29.50 (5.28)	29.20 (4.50)	33.04 (3.75)	17.35	.001	.42
NGSE^2^	20.50 (14.52)	18.20 (3.22)	20.80 (2.34)	22.80	.001	.48

*M = mean; *SD* = Standard Deviation

^1^NGSE—The New General Self-Efficacy Scale

^2^Hope^−^ The Children’s Hope Scale

**Table 4:** Changes in Anxiety levels according to the SCARED sub-scales questionnaire across three times points (N = 50)

	Time 1	Time 2	Time 3			
	M (SD)			F	*p*	Partial Eta Square
Panic Disorder	6.12 (3.96)	5.16 (3.50)	3.38 (2.60)	16.96	.001	.41
Generalized Anxiety Disorder	7.18 (3.61)	6.50 (3.93)	4.60 (3.70)	12.74	.001	.34
Separation Anxiety Disorder	6.94 (2.93)	6.76 (3.03)	4.70 (3.01)	14.78	.001	.38
Social Anxiety Disorder	5.50 (3.74)	5.64 (3.32)	3.74 (3.19)	9.78	.001	.29
School Avoidance	2.40 (3.94)	2.68 (3.29)	2.28 (4.30)	.82	.446	.03
SCARED^1^	26.84 (11.30)	26.02 (11.47)	17.68 (9.98)	20.33	.001	.45

*M = mean; *SD* = Standard Deviation

^1^ SCARED ^–^ Child Anxiety Related Emotional Disorders

A repeated-measures ANOVA was performed to evaluate the effect of time on hope and self-efficacy. Improvement in hope and self-efficacy levels, as evidenced by the Hope and NGSE, were found to be statistically significant (*p* < 0.001). Post-hoc analysis with a Bonferroni adjustment indicated that no significant differences were found between time 1 and time 2. However, post-intervention, at time 3, scores were significantly higher (*p* < 0.001) compared to time 2. High levels of Partial Eta Square indicate a clinical improvement post intervention. See Tables 4.

Follow-up assessments were conducted with 12 participants to evaluate the long-term effects of the ASTride intervention. Results demonstrated improvement in performance across several measures from post-intervention (Time 3) to follow-up (Time 4). Specifically, the BRIEF GEC scores showed a slight improvement, with a mean decrease from 63 (SD = 10.89) at Time 3 to 54.57 (SD = 24.04) at Time 4. However, the change was not statistically significant (Partial Eta Square = .09), indicating only a small effect size. In contrast, Hope scores showed a statistically significant decrease from Time 3 to Time 4. This suggests a decline in hope perception over time.

Similarly, the NGSE scores showed a reduction from Time 3 to Time 4, though the difference was not statistically significant. See Table 5.

**Table 5:** Changes in executive functions, hope and self-efficacy at time 3 compared to follow up at time 4 (N = 50)

	Time 3	Time 4				
	M (SD)		F		*p*	Partial Eta Square
BRIEF^1^; GEC^2^	63 (10.89)	54.57 (24.04)	.626	.45		.09
Hope^3^	33.29 (4.27)	29.8 (5.17)	8.86	.025		.59
NGSE^4^	22.17 (1.16)	19.8 (2.99)	2.50	.175		.33

*M = mean; *SD* = Standard Deviation

^1^BRIEF- Behavior Rating Inventory of Executive Functions; ^2^GEC-Global Executive Composite; ^3^Hope- The Children’s Hope Scale; ^4^NGSE- The New General Self-Efficacy Scale

## Discussion

The present study evaluated the efficacy of the ASTride protocol [14], an EAOT intervention for children diagnosed with ADHD. Our findings demonstrate significant improvements in executive functions, hope perception, and self-efficacy, as well as reduced anxiety levels following the intervention compared to baseline. While behavioral interventions such as Cognitive Behavioral Therapy exhibits improvement in emotional behavioral aspects [26] and cognitive interventions such as Cog-Fun shows improvements in executive functions [8], our results underscore the potential of EAOT as a comprehensive therapeutic approach for addressing the multifaceted challenges associated with ADHD.

Our study aligns with previous research indicating the positive impact of EAS on cognitive and emotional functions. The observed improvements in executive functions, as measured by the BRIEF, are consistent with earlier findings that highlight the benefits of physical activities, including horseback riding, on cognitive performance in children [27]. The significant reduction in the GEC score post-intervention reflects enhancements in key executive functions such as inhibition, working memory, and planning, which are crucial for daily functioning and adaptive behavior [3].

The structured activities in EAS, such as planning and executing tasks related to horse care and riding, provide practical and engaging opportunities for participants to practice and enhance their EF skills and acquire different strategies according to the child's need. The process of learning to communicate and bond with the horse requires sustained attention, adaptability, and emotional regulation. Such activities inherently support the development of cognitive flexibility, as participants must adjust their strategies and behaviors to successfully interact with the animal. Previous studies have shown EAS positively impacts executive functions due to the unique combination of physical activity, therapeutic horse interaction, and the structured, goal-oriented, nature of the therapy sessions [13, 28]

The increase in hope perception and self-efficacy parallels findings from Gulati et al. (2018) who reported enhanced mood and self-esteem in children with ADHD following animal-assisted interventions. Literature indicates that children with ADHD may experience lower levels of hope due to their challenges, which can negatively affect their goal-setting abilities and the identification of strategies to achieve these goals [29]. Nevertheless, recent studies show that hopefulness can enhance executive functions such as planning, flexibility, inhibition, and emotional regulation, even in the presence of ADHD symptoms [30]. Recent findings highlight the importance of positive belief systems, such as hope, in helping children and their parents manage ADHD-related deficits. These beliefs serve as protective factors that contribute to coping strategies and personal goal achievement. It is presumed that during EAS the therapist provides appropriate physical and cognitive challenges that promote a sense of mastery and success, which in turn boost hopefulness and overall well-being.

Our results further contribute to the literature by demonstrating that EAOT can effectively reduce anxiety, as measured by the SCARED. This aligns with studies suggesting that animal-assisted interventions can promote emotional regulation and decrease anxiety through physiological mechanisms, such as reduced cortisol levels and increased oxytocin [31]. It is also hypothesized that the rhythmic movement of the horse can have a calming effect on the rider, which may contribute to the reduction of physiological symptoms of anxiety, such as increased heart rate and muscle tension. This rhythmic motion is thought to stimulate the vestibular system and the body's ability to regulate arousal and maintain a calm state. Furthermore, engaging in horseback riding requires a focus on the present moment and the task at hand, which can divert attention away from anxious thoughts and reduce overall anxiety levels.

In addition to the physical benefits, the human-animal bond plays a crucial role in reducing anxiety. Interactions with horses provide non-judgmental companionship and emotional support [32, 33], which can be particularly comforting for individuals experiencing anxiety. The presence of a horse can serve as a calming influence, helping individuals feel more relaxed and secure. Studies have shown that the social and emotional support provided by horses can decrease feelings of isolation and loneliness [32], often associated with anxiety. ASTride intervention encourages the development of trust, empathy, and social skills, as participants learn to communicate and build relationships with their horse. These positive interactions can lead to increased self-esteem and confidence, further mitigating anxiety. The structured setting of the sessions provides a predictable and safe environment, which is beneficial for individuals who experience heightened anxiety in uncertain or chaotic situations. Further studies may wish to compare between the ASTride protocol and golden standard therapies recommended for children diagnosed with ADHD to examine it's advantages and additional contribution.

Referring to follow-up, only 12 participants participated in the assessments. While improvements were observed post-intervention, some skills appear to diminish over time, suggesting the need for continued intervention to maintain the gains achieved during the study period.

### Limitations and future directions

Despite the promising findings, this study has several limitations. The homogeneity and small sample size, while adequate, limits the generalizability of the results. Future research should include larger, more diverse populations to validate the findings across different demographic and cultural contexts. Additionally, while our study utilized blind assessment methods and standardized valid assessments, potential biases inherent in parental and self-reporting on questionnaires like the BRIEF and SCARED should be considered. Future studies should consider incorporating teachers reports and hands on assessments for a more objective perspective.

This study used an Interrupted Time-Series design, widely recognized as effective quasi-experimental methodology used to assess the effects of interventions and programs implemented in healthcare environments [34]. Future studies may wish to incorporate a randomized controlled trial (RCT) design to assess the ASTride intervention in order to determine whether the observed improvements are directly attributable to EAOT or influenced by external factors. Moreover, while the follow-up assessment at three months post-intervention provides preliminary evidence for the durability of the intervention's effects, our sample is small due to lack of compliance. Longer-term follow-up studies are needed to evaluate the sustainability of the intervention.

## Conclusion

The ASTride EAOT intervention demonstrates significant potential as a comprehensive treatment for children with ADHD, addressing both cognitive and emotional challenges. The findings support the integration of equine-assisted therapies into the broader spectrum of therapeutic options for ADHD, offering a novel, engaging, and effective approach. Further research with more rigorous methodologies and diverse populations will be essential to confirm these findings and establish standardized protocols for widespread application.

## Data Availability

The datasets used and/or analyzed during the current study are available from the corresponding author on reasonable request.

## Abbreviations

ADHD: Attention deficit/hyperactivity disorder
ASTride: Attention skill training (ASTride) protocol
EAOT: Equine-assisted occupational therapy
EAS: Equine-assisted services
BRIEF: Behavior rating inventory of executive function
GEC: Global executive composite
BRI: Behavioral regulation index
MI: Metacognition index
Hope: The children’s hope scale
NGSE: New general self-efficacy scale
SCARED: Screen for child anxiety related emotional disorders
SI: Sensory integration
DSM-5: Diagnostic and statistical manual of mental disorders, fifth edition
RCT: Randomized controlled trial
ANOVA: Analysis of variance
SD: Standard deviation
M: Mean
SPSS: Statistical package for the social sciences
IRB: Institutional review board
